# Systematic literature review of built environment effects on physical activity and active transport – an update and new findings on health equity

**DOI:** 10.1186/s12966-017-0613-9

**Published:** 2017-11-16

**Authors:** Melody Smith, Jamie Hosking, Alistair Woodward, Karen Witten, Alexandra MacMillan, Adrian Field, Peter Baas, Hamish Mackie

**Affiliations:** 1 School of Nursing, The University of Auckland, Private Bag 92019, Auckland, 1142 New Zealand; 2 School of Population Health, The University of Auckland, Private Bag 92019, Auckland, 1142 New Zealand; 3 SHORE and Whāriki Research Centre, School of Public Health, Massey University, Box 6137, Wellesley Street, Auckland, PO New Zealand; 4 Dunedin School of Medicine, University of Otago, Box 56, Dunedin, PO 9054 New Zealand; 5 Dovetail Consulting Ltd, Box 78-146, Grey Lynn, Auckland, PO 1245 New Zealand; 6 Transport Engineering Research New Zealand Limited, Box 11029, Auckland, PO 1542 New Zealand; 7 Mackie Consulting Limited, Box 106525, Auckland, PO 1143 New Zealand

**Keywords:** Walking, Cycling, Health equality, Urban form, Causation, Playgrounds, Walkability

## Abstract

**Background:**

Evidence is mounting to suggest a causal relationship between the built environment and people’s physical activity behaviours, particularly active transport. The evidence base has been hindered to date by restricted consideration of cost and economic factors associated with built environment interventions, investigation of socioeconomic or ethnic differences in intervention effects, and an inability to isolate the effect of the built environment from other intervention types. The aims of this systematic review were to identify which environmental interventions increase physical activity in residents at the local level, and to build on the evidence base by considering intervention cost, and the differential effects of interventions by ethnicity and socioeconomic status.

**Methods:**

A systematic database search was conducted in June 2015. Articles were eligible if they reported a quantitative empirical study (natural experiment or a prospective, retrospective, experimental, or longitudinal research) investigating the relationship between objectively measured built environment feature(s) and physical activity and/or travel behaviours in children or adults. Quality assessment was conducted and data on intervention cost and whether the effect of the built environment differed by ethnicity or socioeconomic status were extracted.

**Results:**

Twenty-eight studies were included in the review. Findings showed a positive effect of walkability components, provision of quality parks and playgrounds, and installation of or improvements in active transport infrastructure on active transport, physical activity, and visits or use of settings. There was some indication that infrastructure improvements may predominantly benefit socioeconomically advantaged groups. Studies were commonly limited by selection bias and insufficient controlling for confounders. Heterogeneity in study design and reporting limited comparability across studies or any clear conclusions to be made regarding intervention cost.

**Conclusions:**

Improving neighbourhood walkability, quality of parks and playgrounds, and providing adequate active transport infrastructure is likely to generate positive impacts on activity in children and adults. The possibility that the benefits of infrastructure improvements may be inequitably distributed requires further investigation. Opportunities to improve the quality of evidence exist, including strategies to improve response rates and representativeness, use of valid and reliable measurement tools, cost-benefit analyses, and adequate controlling for confounders.

**Electronic supplementary material:**

The online version of this article (10.1186/s12966-017-0613-9) contains supplementary material, which is available to authorized users.

## Background

Physical activity is fundamental to human health and wellbeing [1]. Insufficient physical activity is a major contributor to the occurrence of non-communicable diseases and is responsible for about 9% of premature mortality globally [2, 3]. Increasing focus is being placed on the role of the built environment in promoting physical activity, recognising the sustained nature and potential for broad reach of environmental interventions, and the potential to promote substantial changes in population-level physical activity [4]. Active transport also contributes to health-promoting levels of physical activity [5, 6]. In addition, replacing motorised trips with active forms of transportation also brings numerous co-benefits, such as reduced traffic congestion; improved air quality; and reduced fatalities due to traffic, air pollution, and inactivity [7–9]. Accordingly walking and cycling for transport are particular behaviours of interest for researchers and policy-makers alike.

A growing body of evidence shows clear associations between the neighbourhood built environment and physical activity behaviours [10, 11]. Specifically, systematic reviews of the literature have shown that environments characterised as more walkable (i.e., facilitating walking through higher destination accessibility, street connectivity, presence and quality of active transport infrastructure, etc.) are associated with physical activity accumulation for both children and adults [12–27].

McCormack et al. [12] systematically examined the relationship between physical activity and objectively-assessed built environment, improving on earlier reviews by only including cross-sectional studies that adjusted for self-selection, and quasi-experiments. Findings showed land use mix, connectivity and population density and overall neighbourhood design (i.e., walkability, neighbourhood type) were “important determinants of physical activity”. Similarly, Mayne et al. [14] systematically examined the impact of natural or quasi-experimental studies of built environment changes on physical activity. Findings showed greater impacts on physical activity of interventions that were designed to impact active transport. Stronger results were also reported in papers where specific activity constructs (e.g., walking or cycling, rather than total physical activity) were assessed.

Relationships may differ depending on the physical activity construct of interest (e.g., active transport, such as walking or cycling for transport, versus leisure time physical activity) and the type of intervention [12, 28, 29]. In particular, environmental features may be more closely related to active transportation than overall physical activity or recreational activity [12]. Indeed, reviews have consistently shown associations between active transport and built environments that are characterised as “walkable”, including aspects of active transport infrastructure [14, 16, 30, 31]. Less is known with regard to cycling. A systematic review showed cross-sectional associations between cycling and the presence of dedicated cycle routes or paths, separation of cycling from other traffic, high population density, short trip distance, and proximity of a cycle path or green space [18]. For children, an association has been demonstrated between cycling and the promotion of ‘safe routes to school’ [18]. Negative environmental factors were traffic danger, long trip distance, steep inclines and distance from cycle paths. This review also demonstrated some evidence for causality with statistically significant impacts of new cycle routes on cycling prevalence from a limited number of studies [18]. Similarly in their systematic review, Yang et al. [19] observed modest increases in cycling associated with high quality cycling infrastructure improvements.

Relationships may also differ by population group (e.g., children, adults) [15]. Associations for children seem more complex than for adults, with inconsistencies in relationships observed [32], although this may in part be due to the limited evidence base. In their systematic review and meta-analysis of built environments and physical activity in children and youth, McGrath et al. [15] reported adolescents’ physical activity was positively associated with walkability features, play facilities, parks and playgrounds, but these relationships were negative for younger children. Even less is known with regard to older adults, though one systematic review of quantitative and qualitative research reported environmental associates of physical activity in this population, including street lighting, destination accessibility, and pedestrian infrastructure [22].

The quality of the built environment may be an important contributor to health inequalities, especially by influencing opportunities for active transport. To the authors’ knowledge, only one systematic review published to date has considered the impact that sociodemographic factors may have on the efficacy of built environment interventions [25]. In their systematic review, Schüle et al. [25] included only studies that simultaneously considered at least one indicator of “neighbourhood socioeconomic position” (e.g., socioeconomic status, SES) and adjustment for at least one individual socioeconomic factor (ethnicity alone was not considered sufficient) in multilevel modelling of the relationship between the built environment and health outcomes. Almost all the studies in this review showed interactions between “neighbourhood socioeconomic position” and the built environment or individual characteristics, or between the built environment and individual characteristics (including “individual socioeconomic position”). However, substantial heterogeneity in study design and reporting of results hindered any ability to generate clear insights. Hopgood et al. [33] found that traffic calming intensity was more common around less deprived schools in Auckland, New Zealand, whereas Zhu et al. [34] found that schools in Austin, Texas with higher proportions of poor or Hispanic students had better sidewalks and walkability. Interventions to improve the built environment may also have important effects on health equity, for example, improved infrastructure may be used more often by residents with a higher educational or income level [35].

Transport infrastructure is expensive but given the substantial economic burden caused by physical inactivity [3], built environment interventions that help people get active and stay active may be attractive options from a cost-benefit perspective. In terms of benefit:cost ratios, active transport interventions tend to compare well to other major transport investments, such as new roads or public transport [36, 37]. Studies in this field use a range of methodologies and processes to describe and evaluate infrastructural interventions and to model economic outcomes. Even so, findings from a 2008 systematic review reported large positive benefit:cost ratios for walking and cycling infrastructure interventions, with median magnitudes of 5:1 [24]. An updated systematic review reported benefit:cost ratios ranging between −39:1 to 59:1, with positive ratios reported by 26 of the 32 studies included (81%) [27]. More information is needed about specific infrastructural factors at a local level to guide specific investment and planning decisions.

Often, changes in the built environment, experienced either by changing residential location or by intervention in a familiar setting, do not occur in isolation. For example, infrastructural interventions may have associated social media campaigns or supplementary programmes to support behaviour change [38]. It is challenging, yet important, to tease out the effect of built environment changes on physical activity behaviours to enable effective decision making for planning and resourcing environmental change interventions. In one systematic review all included studies that combined built environment and physical activity promotion interventions were reported as being effective in increasing activity, while only half of the built environment intervention only studies showed a positive impact on activity [13]. Only one study in this review was concerned solely with physical activity promotion, limiting comparability between the intervention types.

Notwithstanding frequent calls for research to understand causality [19, 21, 28, 39], until recently, the evidence base has remained predominantly cross-sectional. Likewise, despite calls for studies to improve specificity by examining behaviour-specific environmental attributes and improve objectivity in environmental measures [19, 39], numerous gaps still remain. The lack of evidence has been attributed to gaps in collaboration between disciplines (e.g., research, urban planning) [40], cost of conducting quality research [41], and complexities in evaluating interventions [42] and modelling their effectiveness across population groups [43]. These are substantial barriers, but there has recently been an increase in studies utilising objective and behaviour-specific measures where causality can be inferred (e.g., longitudinal studies, controlled trials).

Some of the difficulties faced in assessing this broad evidence base include variable study quality, insufficient (or no) quality assessment of articles included in reviews [41], a narrow focus on specific population groups or behaviours of interest (restricting understanding from a broader population health perspective), and the predominance of cross-sectional studies (reducing ability to understand causality). There is a dearth of systematic reviews that: (a) consider cost and economic factors associated with built environment interventions, (b) describe socioeconomic or ethnic differences in intervention effects, or (c) where the ability to isolate the effect of the built environment from other intervention types is assessed.

For these reasons, we believe it is timely to re-examine the evidence base, with a focus on updating and improving on previous reviews, by: (1) only including studies where causality can be implied, (2) considering intervention cost, (3) examining whether intervention effects differ by ethnicity or SES, (4) conducting rigorous article quality assessment, (5) including all age groups and physical activity behaviours, and (6) attempting to isolate the effect of the built environment from other interventions occurring in studies. Our aims are to: (1) conduct a new systematic review to identify which environmental interventions increase physical activity in residents at the local level, with the goal of informing future policy and practice in community design; and (2) to build on the limited evidence base on the effectiveness of built environment interventions for influencing health inequalities by systematically exploring the effectiveness of these interventions by ethnicity and SES.

## Methods

The review protocol was prepared following the PROSPERO International prospective register of systematic reviews protocols [https://www.crd.york.ac.uk/prospero/] and published on figshare [44].

### Eligibility criteria

Articles were eligible if they reported a quantitative empirical study (natural experiment or a prospective, retrospective, experimental, or longitudinal research (including repeated cross-sectional surveys)) investigating the relationship between objectively measured built environment feature(s) and physical activity and/or travel behaviours in children or adults. Qualitative studies, or those that did not measure change in both the independent and dependent variables were excluded. For trials, no control group was required for the study to be included in the review.

### Information sources

A systematic search of Scopus, Ovid (all journals), ProQuest Science, ProQuest Social Science, and the Transport Research International Documentation database (comprising the US Transportation Research Board’s Transport Research Information Services database and the Organisation for Economic Co-operation and Development Joint Transport Research Centre’s International Transport Research Documentation database), was conducted by the lead author (MS) in June 2015. Databases were identified in consultation with a subject-specific librarian, and the wider research team comprising specialists in transport, built environments, physical activity, active transport, epidemiology, and health. We acknowledge the value of grey literature in some circumstances but did not include studies reported in this form in this study. We sought the most robust evidence available, ensuring our methods were as robust and replicable as possible [45], and reducing the risk of bias in outcome reporting that may occur in the grey literature, for instance in settings where funding for infrastructural work is at stake. Bibliographies of included articles were also searched for possible relevant articles (using the article title).

### Search strategy

Keyword searches of article abstracts and titles were conducted using three categories: 1) environments, 2) physical activity or travel modes, and 3) natural experiments, or prospective, retrospective, experimental, or longitudinal. Search terms were identified from MeSH subject headings in PubMed, previous similar reviews [5, 12, 46], and the knowledge and expertise of the research team. Test searches were conducted to gauge the sensitivity and specificity of the search terms, and amendments were made accordingly. Searches were limited to English-language articles that were published or in press, with no date restrictions. The final search strategy is outlined in Additional file 1.

### Study selection

Titles and abstracts of articles were screened by the lead author and included if they met the eligibility criteria. Where it was unclear whether articles met the inclusion and exclusion criteria from the abstract and title, full-text articles were sourced. Where bibliography searches identified article titles as possibly relevant, article abstracts were sourced and screened using the above criteria. All processes (i.e., identification of articles, data extraction, quality assessment) were duplicated by a co-author (JH) with a random selection of 10% of each dataset [47, 48]. Any disagreement was resolved through discussion, and any necessary amendments made to each process.

For the purposes of this examination, a relatively broad definition of built environment was used, with the aim of identifying and understanding the range of modifiable factors in the external neighbourhood environment that may impact people’s physical activity or travel behaviours. Accordingly, ‘built environment’ encompassed either interventions or changes occurring at the individual level (e.g., due to moving), or at the local, neighbourhood, or town scale. Measures were all objective and included geographic information systems-derived variables (e.g., dwelling density, distance to destinations), community infrastructural or streetscape intervention typologies (e.g., shared spaces, naked streets), natural or built aesthetic factors in the neighbourhood environment (e.g., tree planting, signage, wayfinding), and measures of other relevant environmental supports for physical activity or active travel (e.g., playground features). Recognising the contribution that public transport use can make to physical activity accumulation [49, 50], studies assessing changes in access to public transport (e.g., distance to closest public transport stop, park-and-ride interventions) were also eligible. As the focus was on being able to isolate the effect of built environment features or interventions, studies investigating aggregate measures (e.g., walkability, walk score), or studies combining infrastructural and “soft” (e.g., awareness programmes, social media, organised programmes) interventions where the effect of the infrastructural intervention could not be isolated were not included. Conversely, studies that included infrastructural and soft interventions but where findings enabled the effect of the infrastructural intervention to be isolated were included.

Likewise, a broad approach was taken to defining physical activity for this review – encompassing all types and dimensions of self-reported or objectively assessed physical activity (e.g., recreational walking, habitual physical activity, moderate-to-vigorous physical activity). Physical activity could be assessed retrospectively, prospectively, using repeated cross-sectional surveys or direct observation, or before and after an intervention. While acknowledging the limitations of recall, well-designed retrospective studies offer a “plausible alternative to prospective longitudinal data collection” [51] due to time efficiency, lack of panel attrition, cost effectiveness, and quality of survey instruments (as earlier measures may be obsolete).

### Data extraction and quality assessment

A study-specific data extraction form (available in Additional file 2) was generated from a previous systematic review of health impacts of new roads [52] and the Effective Practice and Organisation of Care data collection form [53]. Data were extracted for all included articles across seven categories: general information, population and setting, methods, participants, intervention groups, outcome measures, and results. In addition, data were extracted on whether the effect of the built environment differed by ethnicity or SES. Studies were eligible for the latter if they reported effect estimates stratified by ethnicity or SES, or examined effect modification/interactions between the intervention or exposure variable and ethnicity or SES. Eligible measures of ethnicity included self-reported or objectively assigned ethnicity or race. Eligible measures of SES included income, educational level, occupation and home ownership, as well as composite indices such as those for deprivation. SES could be measured at an individual or area level. Area-level SES measures were only eligible if the SES variable was calculated for a smaller area than the study/intervention area (e.g., meshblock-level deprivation [54] calculated in a community-wide project that included multiple meshblocks).

Strength of evidence was determined using the *Evaluation of Public Health Practice Projects Quality Assessment Tool* (EPHPP) as employed in previous similar reviews [5, 46], and adapted to improve the suitability for assessing articles included in this review [46] as outlined in Additional file 3. EPHPP scoring criteria are provided in Additional file 4. Adaptations were made as a consequence of the duplicate quality assessment process whereby the reviewers identified a number of necessary clarifications to facilitate consistency in scoring. These changes did not impact the final quality assessment scores.

### Summary measures, synthesis of results and quality assessment/risk of bias across studies

The key outcomes of interest were physical activity (self-reported, observed, or objectively assessed), active transport (self-reported or observed), and visitation to or use of a setting (e.g., counts of riders on new cycleways; counts of playground users). Findings were collated for each of these outcome measures and considered separately by population subgroups (children, adults). Findings were also summarised in the context of study quality to gauge risk of bias and understand the strength of evidence provided.

## Results

**Fig. 1:**
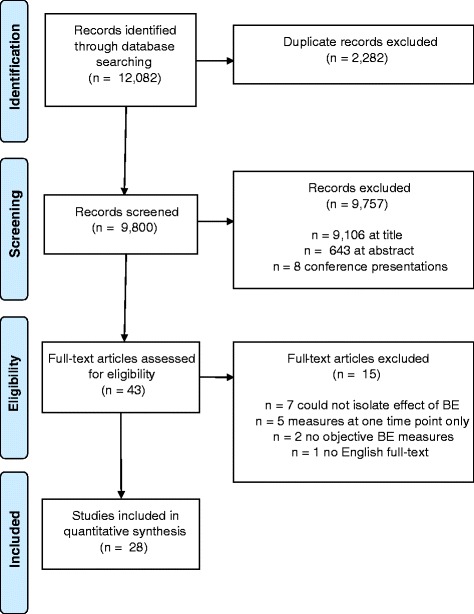
Pathway of articles included and excluded in review

Figure 1 provides the flow diagram of articles included and excluded from the review [55]. Of the 12,082 articles identified, 2282 were duplicates, 9757 were excluded at title or abstract stage, and 43 were assessed at full text stage. In total, 28 articles met the inclusion criteria for this review.

### Study characteristics

Key characteristics of studies included are provided in Table 1. The majority of studies were controlled repeat cross-sectional examinations (*n* = 8), followed by uncontrolled repeat cross-sectional studies and uncontrolled longitudinal studies (both *n* = 6). Almost three-quarters (71%) of studies were conducted in the USA, with the remainder from Australia (*n* = 4), and Belgium, England, Scotland, and New Zealand (all *n* = 1). A majority of studies focused on all age groups (*n* = 13) or adults only (*n* = 12). Three (11%) focused on children and no studies focused specifically on older adults. Intervention types varied widely, but predominantly involved infrastructural interventions for facilitating walking and cycling (e.g., bicycle boulevards, installation of cycle lanes, improving sidewalks, etc.). Park and playground improvements or development also featured regularly. Walking for leisure or transport was assessed in seven studies, cycling in two, and overall active transport in six studies. Where observation of active transport occurred, two studies directly observed cyclist counts only, and two undertook counts of pedestrians and cyclists.

**Table 1:** Characteristics and key findings of studies included in review

Author; year of publication; study location	Population description	Study design	Intervention description	Intervention delivery	Physical activity outcome	Key findings
Beenackers; 2012; Perth, Australia [70]	1427 adults participating in the RESIDE study - movers to one of 74 new residential housing developments in Perth	Uncontrolled longitudinal natural experiment	Change in objectively assessed built environment from original household to new residence. Objective neighbourhood variables were constructed in GIS using a 1600-m network service area buffer around the residential address. Street connectivity, residential density, land-use mix, and number of destinations relevant for transport or recreation were calculated.	Mechanism: moving from one neighbourhood to a new development. Medium: Liveable neighbourhood design, focusing on integrated mixed use and interconnected network.	Cycling for transport or recreation	After full adjustment increased residential density was associated with increased odds of taking up cycling for transport (OR 1.54, 95% CI 1.04, 2.26, *p* = 0.03), and increased street connectivity was associated with increased odds of taking up recreational cycling (OR 1.20, 95% CI 1.06, 1.35 p < 0.01).
Boarnet; 2005; California, USA [85]	Children attending one of nine intervention schools (3rd-5th grade), *n* = 1778 at baseline	Uncontrolled cross-sectional pretest-posttest	Types of intervention treatments investigated: sidewalk improvement projects (e.g., install pathway and signage; install sidewalk gap closures; install sidewalk, curb, gutter); traffic signal improvements (install traffic signal to replace four-way stop sign); crosswalk and crosswalk signal improvements (install in-pavement crosswalk signal system; install pedestrian-activated flashing warning system; add pedestrian count-down signals; install crosswalk and crosswalk signs); Install bike lanes.	Mechanism: school-neighbourhood infrastructural interventions. Medium: sidewalk improvements, traffic signal improvements, crosswalk improvements, bicycle path improvements.	Walking and cycling to and from school	Sidewalk, traffic signal, and crosswalk improvements were associated with greater increases in walking for children who passed the intervention on the way to school compared with those who did not (differences of 11.59%, *t* = 3.01; 15.63%, *t* = 2.43, and 21.16%, *t* = 3.15 across the three areas where gap closures were implemented; 11.34%, *t* = 2.12 for new sidewalks plus sidewalk gap closures; 6.67%, *t* = 1.04 for new pathway of decomposed granite; 10.94%, *t* = 2.80 and 14.43%, *t* = 2.52 for traffic signals replacing 4-way stop sign; 3.13%, *t* = 1.02 and 4.31%, t = 2.52 for in-pavement crosswalk lighting; and 13.33%, *t* = 1.85 for pedestrian count-down signals). There was insufficient evidence to determine effectiveness of the one bicycle path improvement project.
Brown; 2008; Salt Lake City, Utah, USA [86]	102 adult residents of households within one half mile of a new light rail stop	Uncontrolled longitudinal pretest-posttest	Intervention was a new light rail stop on the Salt Lake City TRAX line.	Mechanism: infrastructural interventions. Medium: increase in public transport accessibility	Moderate intensity activity bouts lasting at least 8 min per valid hour; number of leisure walks in last 2 weeks	Adjusted for income and employment, respondents who were rail users at baseline and follow-up had the largest number of moderate physical activity bouts (mean [SD] 3.68 [0.60] compared with 1.77 [0.83] for new riders at followup, and 1.07 [0.76] for non rail users at baseline and followup, F for ridership group effects = 3.89, *p* = 0.03). No difference observed between groups for leisure walks.
Clark; 2014; Southern Nevada, USA [61]	All users of intervention and comparison trails, *n* = 191 at baseline	Quasi-experimental cross sectional pretest-posttest	Intervention: 6 community trails which included a marketing campaign promoting trail use and the addition of way-finding and incremental distance signage to selected trails (October 2011–October 2012). The distance markings were embossed into the surface of the trails at 0.25 mile intervals. Way-finding signs were placed on the trails at major access points, as suggested by the local jurisdictions, and were mounted on square metal posts. Each side of the post was marked with a trail map, the name of the trail, the logo of the responsible jurisdiction, and icons for acceptable and unacceptable uses.	Mechanism: infrastructural interventions. Medium: presence of additional features.	Counts of trail users	Increases in mean users per hour were found for both the intervention and control trails (increased by 31%, *p* < 0.01 and 35%, p < 0.01, respectively). Increases did not vary between the treatment groups (Z = 0.9892, *p* = 0.32).
Cohen; 2012; Los Angeles, California, USA [57]	Users of 12 parks receiving outdoor exercise equipment interventions, and users of ten comparison parks, *n* = 7105 observed and *n* = 742 surveyed at baseline	Controlled cross-sectional pretest-posttest	“Fitness Zones” were implemented in 12 parks - comprising durable, weather and vandal resistant exercise equipment for strength training and aerobic exercise. Eight pieces of equipment were installed in each park	Mechanism: infrastructural interventions. Medium: provision of new equipment	Number of exercise sessions per week, active transport to park, METs	Intervention respondents reported engaging in more exercise sessions per week than comparison park respondents (2.9 versus 2.7; *p* < 0.0001) and getting to the park more often by walking than comparison park respondents (56% versus 35%, *p* = 0.002). A net gain of 1909 METs was observed in the 12 intervention parks, but this increase was not significant.
Cohen; 2014; Los Angeles, California, USA [87]	Users of pocket and comparison parks; 392 adults aged 18 years or older residing in houses within 0.5 miles of control and intervention parks	Controlled cross-sectional pretest-posttest	Three pocket parks were developed, two in previously vacant lots and the third in a former community garden site. All three had playground equipment and benches installed, and a walking path was developed around the largest park. All were fenced and enclosed by lockable gates.	Mechanism: development of new activity setting. Medium: retrofitting of existing green spaces to park settings, including installation of playground equipment	METs, MVPA, park visitation, leisure time exercise	At follow up, 25% of park users were engaged in MVPA in pocket parks compared with 36–41% in comparison parks. Estimated METs during observation times at follow up for pocket parks was 324, compared with 280–526 in comparison parks. Survey results showed a threefold increase in visits to any park at least once a week for residents living in pocket park areas (11.1% at baseline versus 33.9% at followup, *p* < 0.0001), an increase in engaging in leisure time exercise (25.8% versus 35.7%, *p* = 0.0025), and an increase in exercising in the park (9.6% versus 14.4%, *p* = 0.0395).
Cohen; 2015; San Francisco, USA [88]	Users of control and intervention parks; 922 residents of houses within 0.5 miles of control and intervention parks	Controlled cross-sectional pretest-posttest	Two parks underwent extensive renovations including installation of new play equipment, landscaping, and ground surfaces. One had added adult outdoor fitness equipment and a new recreation centre. Two parks were partially renovated and intervention components were not described.	Mechanism: infrastructural interventions. Medium: provision of new equipment and improved aesthetics and surfaces	Park use and METs expended in the park; frequency of park visitation; weekly exercise sessions	Increases in observed park use and METs occurred for the two parks with completed renovations only (233% and 255% respectively, both *p* < 0.001) versus decreases of 49% and 53% respectively in the control parks both *p* < 0.001), and no significant change for the parks under construction. Self-reported frequency of visitation increased for the control and renovated parks range 0.22–0.73, *p* < 0.01), and a significant decrease in visitation was reported for partially renovated parks (by approximately half, *p* < 0.001). The number of weekly exercise sessions increased for survey respondents in the partially renovated park areas only (0.29, p < 0.01), and decreased for respondents of park intercept surveys in renovated parks only (−0.42, p < 0.001)
D’Haese; 2015; Ghent (Flanders), Belgium [66]	167 children aged 6–12 years and their parents	Controlled longitudinal pretest-posttest	Twelve Play Street projects that lasted at least seven consecutive days were included in this study. A Play Street is organized between 1400 and 1900. The city council of Ghent also offers a box with play equipment that can be hired for free by the volunteers of the Play Streets. They can keep the box during the period of the Play Street intervention.	Mechanism: At least three volunteers living in the street have to sign an agreement with the city council to hold responsibility for the organization of the Play Streets. They are the contact persons between the city council and the other street inhabitants. The task of the volunteers is to inform the street inhabitants about the rules and timing of the Play Streets. The fences and traffic signs are delivered by the city council. Parents remain responsible for their children playing in the street. Medium**:** Each day the Play Street is organized, the volunteers enclose the Play Street with fences and a traffic sign, indicating that car traffic is forbidden in the streets. The council also offers a box with play equipment that can be hired for free and kept during the intervention period. Other larger materials are also available for free hire for one day. Inhabitants may also organise their own activities.	MVPA	Positive intervention effect was found for MVPA (β = 0.82 ± 0.43;χ2 = 3.6; *p* = 0.06). Between 1400 and 1900, MVPA of children living in Play Streets increased from 27 min during normal conditions to 36 min during the Play Street intervention, whereas control children’s MVPA decreased from 27 to 24 min. The intervention effects on MVPA (β = −0.62 ± 0.25; χ2 = 6.3; *p* = 0.01) remained significant when the effects were investigated during the entire day, indicating that children did not compensate for their increased MVPA during the rest ofthe day.
Dill; 2014; Portland, Oregon, USA [62]	490 adults residing within 1000 ft of intervention or control streets	Controlled longitudinal pretest-posttest	Bicycle boulevard installation in eight street segments (0.9–4.2 miles long).	Mechanism: Improved infrastructure for cycling. Medium: Installation of bicycle boulevards (0.9–4.2 miles long).	MVPA, active transportation	A significant decrease in number of minutes biked was observed in the intervention neighbourhood (where biking occurred for at least 10 min; β −0.09, p < 0.01). No other differences were observed for MVPA, walking, or biking (number of bike trips, made a bike trip, made a bike trip greater than 10 min duration).
Fitzhugh; 2010; Knoxville, Tennessee, USA [89]	Children, adolescents, and adults in free-living conditions within one experimental and two control neighbourhoods	Controlled cross-sectional, pretest-posttest	Retrofıtting a neighbourhood with an 8-ft-wide and 2.9-mile-long asphalt urban greenway/trail to connect the pedestrian infrastructure with nearby retail establishments and schools.	Mechanism: transport and recreation setting infrastructural interventions. Medium: new urban trail for walking and cycling.	Physical activity and active transport measured in the neigbhourhood and around schools (for school travel behaviours)	Total physical activity increased in the experimental neighbourhood only (median increase of 8 counts at follow-up, *p* < 0.001), and was greater in the experimental neighbourhood compared with the control neighbourhood at followup (median difference of 12 counts, *p* = 0.028). Changes in neighbourhood level walking (*p* < 0.001) and cycling (*p* = 0.038) were greater in the experimental neighbourhood than the control neihbourhood. There was no difference over time for the experimental neighbourhood for active transport to school, or for the difference in change in active transport to school between the experimental and control neighbourhoods (*p* = 0.2061).
Giles-Corti; 2013; Perth, Australia [69]	1808 adults (mean age 40.7 years) participating in the RESIDE study - movers to one of 74 new residential housing developments in Perth	Uncontrolled, longitudinal, repeated measures	At each time point, objective built environment measures were generated using geographic information systems. These measures included (standardized) neighbourhood walkability measures, such as street connectivity, residential density, and land-use mix, that were calculated for the areas accessible along the street network within 1600 m from the participants’ home.	Mechanism: Neighbourhood walkability. Medium: Street connectivity, residential density, land-use mix, number of bus stops, presence of railway station, number of types of services, number of types of convenience stores, number of types of public open spaces	Walking for transport or recreation	In fully adjusted models, transport-related walking increased by 5.8 min per week for each type of transport-related destination that increased (*p* = 0.045) and recreational walking increased by 17.6 min per week for each type of recreational destination that increased (*p* = 0.070). The association between the built environment and recreational walking was partially mediated by changes in perceived neighbourhood attractiveness: when changes in ‘enjoyment’ and ‘attitude’ towards local walking were removed from the multivariate model, recreational walking returned to 20.1 min/week (*p* < 0.040) for each type of recreational destination that increased.
Goodman; 2014; Cardiff, Kenilworth, and Southampton, UK [56]	3516 adults aged 18–89 residing within 5 km of the core Connect2 intervention	Uncontrolled, longitudinal, repeated measures	Cardiff: a traffic-free bridge was built over Cardiff Bay; Kenliworth: a traffic-free bridge was built over a busy trunk road; Southampton: informal riverside footpath was turned in to a boardwalk.	Mechanism: Major infrastructural interventions for improved walking and cycling. Medium: construction of bridges and boardwalks for non-motorised traffic	Use of the new infrastructure; self-reported walking or cycling for transport or recreation; self-reported time spent in moderate intensity leisure time physical activity and vigorous intensity leisure time physical activity	Overall 32% and 38% of participants reported using the infrastructure at years 1 and 2, respectively. Little evidence was found for whether proximity to the intervention predicted changes in the activity levels of residents at the year 1 follow-up. At year 2, individuals living closer to the intervention reported significant increases in walking and cycling relative to those living farther away (an effect of 15.3 min per week per km closer to the intervention; 95% CI 6.5, 24.2 min per week). Total recreational physical activity increased more for individuals living closer to the intervention than those living further away at 2 years (β = 12.5, 95% CI 1.9, 23.1).
Gustat; 2012; New Orleans, USA [63]	499 adults aged 18–70 residing in low-income, primarily African American neighbourhoods receiving infrastructural interventions and two comparison neighbourhoods	Controlled cross-sectional pretest-posttest	Path intervention neighbourhood: addition of an 8-ft-wide path of 6 blocks on a grassy, tree-filled median of a wide neighbourhood boulevard. The path connected a park outside the intervention area to a commercial corridor. Playground intervention: installation of a playground on the back lot of a local elementary school. The project paid supervisors to keep the fenced playground open after school hours and on weekends from summer 2007 through spring 2009.	Mechanism: site-specific infrastructural interventions. Medium: major streetscape improvements for walking and cycling and installation of a new playground accessible to the community outside school hours	Walking for transportation or leisure; MVPA	No difference in walking for transportation or leisure between control and intervention. Increase in proportion of people engaged in MVPA and vigorous activity only in the intervention area with the new path compared with the two control neighbourhoods (increase to 41% at followup compared with decreases to 24% and 38%, p < 0.001 for MVPA and increase of 3.2% compared with decreases in all other neighbourhoods, p < 0.001 for vigorous physical activity)
Harduar-Morano; 2008; Florida, USA [90]	243 adult residents of households in the intervention community, aged 18–64 years	Retrospective, uncontrolled	Community-wide improvements, including activity-specific improvements (streetlights installed and creating safe places to walk and exercise outdoors).	Mechanism: community-wide infrastructural interventions. Medium: installation of streetlights, creating safe places to walk and exercise outdoors	Exercising outside more than 2 years ago	Sixty-three percent of survey respondents indicated that improvements were made on streetlight installation and the creation of safe places to walk and exercise outdoors, which are both community improvement issues related to outdoor activities. Of these respondents, 95% provided a positive response when asked if they spent more time exercising outside than two years ago. When the issues related to outdoor activities were examined separately, respondents were more likely to exercise outdoors now compared to two years ago if they also indicated that the following issues had improved (vs. no change): installation of streetlights (OR = 18.33; 95% CI = 4.22, 79.64) or the creation of safe places to walk or exercise outdoors (OR = 14.18; 95% CI = 3.94, 51.03). Overall, 80% agreed they spent more time exercising outside than 2 years ago “because of the newly installed streetlights, park exercise equipment, and sidewalks”
Knuiman; 2014; Perth, Australia [71]	1813 adults (average age 39 years) participating in the RESIDE study - movers to one of 74 new residential housing developments in Perth	Uncontrolled, longitudinal, repeated measures.	At each time point, objective built environment measures were generated using geographic information systems. These measures included (standardized) neighbourhood walkability measures, such as street connectivity, residential density, and land-use mix, that were calculated for the areas accessible along the street network within 1600 m from the participants’ home.	Mechanism: Neighbourhood walkability. Medium: Street connectivity, residential density, land-use mix, number of bus stops, presence of railway station, number of types of services, number of types of convenience stores, number of types of public open spaces	Walking for transport	Both the connectivity and land-use mix walkability components (but not residential density), as well as neighbourhood access to public transit, were significantly related to transport walking in the neighbourhood in the fully adjusted model with fixed effects. Connectivity had an estimated subject-level OR of 1.13 (95% CI 1.01, 1.26), land use mix had an estimated subject-level OR of 1.33 (95% CI 1.16, 1.52). Participants who had 30 or more bus stops within 1600 m of their homes had odds of walking for transportation that were approximately double those of participants who had 0–14 bus stops, and the presence of a train station within 1600 m increased the odds of walking for transportation by approximately 50%. The objectively measured number of types of destinations was significantly related to walking for transportation in a dose-response manner (p for trend <0.05); however, when categorized into levels, the ORs comparing neighbourhoods with 8–15 destinations types within 1600 m to those with 0–3 was only approximately 1.3 (*p* = 0.04).
Lott; 1979; California, USA [91]	3364 cyclists in the intervention street and two parallel control streets	Controlled cross-sectional, pretest-posttest	Intervention involved installing bicycle lanes on both sides of the street. Control streets had existing bicycle lanes, but no description of these provided.	Mechanism: Improved facilities for cycling. Medium: Addition of new bicycle lanes.	Cycling	Significant increases in cyclists were observed in the intervention street compared with the control street, for adults aged 25 years and older only (x^2^ = 3.20, *p* = 0.08).
McDonald; 2013; Eugene, Oregon, USA [64]	Children attending one of 9 intervention or 5 control schools (K-8).	Controlled cross-sectional, uneven repeated measures	Groups: Education/Encouragement only: Education and crosswalks/sidewalks; Education and boltage; Education and covered bike parking; Education and covered bike parking and (crosswalks/sidewalks or Boltage). Boltage is a program which encourages biking by tracking frequency of biking to school and offering prizes based on participation.	Mechanism: school-neighbourhood infrastructural interventions. Medium: provision of improved and covered bike parking, crosswalk and sidewalk improvements.	Walking or cycling to school	Augmenting education programs with additional infrastructural improvements was associated with increases in walking and biking (only for education plus covered bike parking) of 4–18 percentage points above that gained from education/encouragement only (*p* < 0.05). Changes for education only were significant for proportion biking (β = 0.699, marginal effect [discrete change in outcome for each observation averaged over the sample, weighted by number of students surveyed] = 0.050 95% CI 0.019, 0.080) but not walking. Changes for education plus covered bike parking were β = 1.094, marginal effect = 0.188, 95% CI 0.095, 0.281 for walking, and β = 1.211, marginal effect 0.106, 95% CI 0.018, 0.195.
Morrison; 2004; Glasgow, Scotland [92]	244 adult residents of households within the intervention neighbourhood aged 15+ years	Retrospective (uncontrolled longitudinal pretest-posttest but physical activity outcome was retrospective report at follow-up)	The traffic calming scheme was built in the main road bisecting a deprived urban housing estate in Glasgow, Scotland. The scheme comprised five sets of speed cushions (raised platforms on the road to slow car drivers), two zebra crossings with adjacent railings, and creation of parking bays.	Mechanism: infrastructural interventions. Medium: presence of traffic calming features.	Walking or cycling in the area more	After the introduction of the traffic calming Scheme 20% (95% CI 14.1, 25.9) of respondents said that they walked in the area more as a result of it, and 3.8% said they cycled in the area more because of the scheme (95% CI 0.8, 6.8). With the exception of pensioners on one stretch of the road, the pedestrian count recorded substantial increases at most sites and in most age groups after the traffic calming scheme was built.
Parker; 2011; New Orleans, USA [58]	All users of a newly constructed bike lane	Uncontrolled cross sectional pretest-posttest	A 3.1 mile dedicated bike lane was installed. Bike lanes were striped on both sides of the road and were 5 ft wide.	Mechanism: New infrastructure for cycling. Medium: installation of dedicated bike lanes	Number of cyclists	A 57% increase in the average number of riders per day was observed; there was a 133% increase among adult female riders, and a 44% increase in adult male riders (all *p* < 0.001).
Parker; 2013; New Orleans, USA [93]	All users of a newly constructed bike lane and cyclists in two adjacent streets	Uncontrolled cross sectional pretest-posttest	A 1 mile dedicated bike lane was installed. Bike lanes were striped on both sides of the road and were 5 ft wide.	Mechanism: New infrastructure for cycling. Medium: installation of dedicated bike lanes	Number of cyclists	Mean number of cyclists increased significantly on the intervention street (average of 79 cyclists at baseline; 257 at follow-up), and decreased on the side streets (average of 54 at baseline; 36 at follow-up; location by time interaction Z = 24.27, p < 0.001). The increase in cyclists was greater among females than males.
Quigg; 2011; Dunedin, New Zealand [65]	184 children aged 5–10 years attending schools within the intervention and control neighbourhoods.	Controlled, longitudinal pretest-posttest	Playground upgrades in two playgrounds in the intervention community. In one playground, ten new components (including play equipment, seating, safety surfacing) were installed, and two existing components were removed. At the second playground, two new play equipment pieces were installed, and a small modification was made to another piece of equipment.	Mechanism: Improved quality of neighbourhood playgrounds. Medium: Addition of equipment, seating, and safety surfacing, removal or modification of existing equipment.	Total daily physical activity	Change in physical activity was associated with an interaction between body size (body mass index z-score) and intervention status (*p* = 0.006) whereby greater increases in activity were observed in the intervention neighbourhood for children with lower body mass index scores, but lower levels of activity for children with higher body mass index scores.
Ranchod; 2013; Multiple sites across USA [59]	6191 adult participants aged 45–84 from the Multi-Ethnic Study of Atherosclerosis, aged 45–84 years and free of clinical cardiovascular disease at baseline	Uncontrolled, longitudinal, repeated measures.	At each time point, an objective measure of recreation facility density was calculated using geographic information systems	Mechanism: Neighbourhood recreation facility density. Medium: Density of recreational facilities in individual participants’ neighbourhoods	Recreational physical activity	A greater increase in recreational density was associated with a less pronounced decline in physical activity (mean difference in annual change in physical activity for each 1-unit increase in recreational density over time = 10.3 (95% CI 0.7, 19.9). The association was stronger in older adults.
Roemmich; 2014; Grand Forks, North Dakota, USA [94]	245 users of the intervention setting (playground within a park) except teenagers aged 13–18. Classified as child (0–12 years), or adult (19+ years)	Uncontrolled cross-sectional pretest-posttest	Removal of seating around a playground. A-B-A design, where seating was reinstalled after the intervention period.	Mechanism: Community park infrastructural interventions. Medium: removal of seating around a playground	MVPA, park visitation	Physical activity intensity for children, adults, and families (combined children and adults) was significantly greater (*p* < 0.02) during the intervention period than for the pre or post measurements. In the first study The ORs of adults being in MVPA rather than sitting during the intervention period compared with the pre measurement and post measurement were 4.1 (95% CI 1.1, 15.1, *p* < 0.03) and 22.7 (95% CI 4.2, 122.0, p < 0.001), respectively. For children, the odds of being in MVPA rather than sitting were not associated with study condition (*p* ≥ 0.45). Significant differences in visitation were observed, with the intervention condition associated with lower visitation than other conditions (*p* < 0.0001)
Shu; 2014; Santa Monica, California, USA [95]	Users of Ocean Park Boulevard (intervention site)	Uncontrolled cross sectional pretest-posttest	Major street upgrade on a 1 km section of Ocean Park Boulevard. The segment of urban residential roadway originally featured raised sidewalks, dedicated on-street parking and bicycle lanes, and one vehicle lane in each direction. The intervention involved widening sidewalks, adding street furniture and over 100 new trees; improving existing crosswalks and bicycle lanes with more clearly marked flashing beacons and asphalt painting; raising and adding trees to the centre median; adding 75 pedestrian-scaled light poles were added; and many other improvements were made (e.g., storm-water management).	Mechanism: street-level infrastructural interventions. Medium: major streetscape improvements for walking and cycling.	Traffic volume of pedestrians and cyclists	The number of pedestrians increased by 37% compared to pre-retrofit conditions and the number of cyclists remained approximately the same. Except for the weekday morning session, all session-average increases were significant (*p* < 0.05).
Tester; 2009; San Francisco, CA, USA [96]	All users of two intervention parks and one control park setting, *n* = 1006 at baseline	Uncontrolled cross-sectional pretest-posttest	Artificial turf replaced uneven dirt fields, and new fencing, landscaping, lighting, and picnic benches were added. One park additionally had ‘soft’ programme initiatives such as professional training and skills development for park and recreation staff and expanded programs driven by community input.	Mechanism: Community park infrastructural interventions. Medium: improvements and additions to park infrastructure (turf grass, lighting, fencing, etc.)	Number of park users, proportion of users participating in MVPA	Average number of park users increased significantly across all age groups (*p* ≤ 0.008) except for teenage boys in both intervention parks (*p* = 0.813 and *p* = 0.931), and except for teenage girls only for the infrastructural plus programme intervention park (*p* = 0.116). Increases were more pronounced in the infrastructure plus programme park for children and adults, and the infrastructure intervention only for seniors. Mean number of people classified as moderately or vigorously active increased in both intervention parks (range of 0.29 for female vigorous activity in the infrastructure only park to 7.28 for male moderate activity in the infrastructure plus programme park; all *p* ≤ 0.05). With the exception of an increase in mean number of males classified as moderately active (increase of 2.39, *p* = 0.01), no changes in physical activity were observed for the control park.
Veitch; 2012; Melbourne, Victoria, Australia [97]	All park users aged 2 or more years, *n* = 235 at baseline	Controlled cross-sectional pretest-posttest	A park that was originally an open space area with few amenities. The intervention involved the establishment of a fenced leash-free area for dogs, an all-abilities playground, a 365 m walking track, a barbecue area, landscaping, and fencing to prevent motor vehicle access to the park.	Mechanism: infrastructural interventions. Medium: presence of additional features.	Walking and vigorous physical activity, number of park users	Significant difference in change in number of observed park users between the intervention park (increased from 235 to 985) and control park (decreased from 83 to 51, F = 14.99, *p* < 0.0005). Significant difference in change in number of people observed walking (increased from 155 to 369 in intervention park and decreased from 75 to 51 in control park, F = 11.70, p < 0.0005). Significant difference in change in counts of people being vigorously active (increased from 38 to 257 in intervention park, decreased from 5 to 0 in control park, F = 4.98, *P* = 0.008).
West; 2011; USA [98]	368 adult residents aged 30+ years living within 0.5 and 0.51–1.0 miles of a newly installed greenway (the intervention)	Uncontrolled longitudinal pretest-posttest	5 miles of greenway were developed and added to an existing greenway (an open space corridor reserved for recreational use or environmental preservation that connects urban centres).	Mechanism: recreation setting infrastructural interventions. Medium: additional greenway space for walking and cycling.	Walking, MVPA	Number of days spent walking increased for all respondents (by a mean of 0.48 days for residents living ≤0.50 miles to the new greenway section and 0.26 for those living 0.51–1.0 miles from the greenway). No significant interaction effect for time and proximity to the intervention was observed (*p* = 0.363). Number of days participating in MVPA increased for all respondents (by a mean of 0.63 days for residents living ≤0.50 miles to the new greenway section and 0.48 for those living 0.51–1.0 miles from the greenway). No significant interaction effect for time and proximity to the intervention was observed (*p* = 0.476). Number of days participating in vigorous activity increased by 0.46 days for residents living nearer and further from the intervention; no significant interaction effect for time and proximity to the intervention was observed (*p* = 0.962).
West; 2015; North Carolina, USA [60]	524 property owners who owned a single-family dwelling unit valued at more than $5000 and located within 1 mile of the greenway intervention or in the control area	Controlled longitudinal pretest-posttest	1.93 miles of greenway were developed and added to an existing greenway.	Mechanism: New infrastructure for walking and cycling. Medium: addition of 1.93 miles of greenway to an existing greenway	Walking, moderate physical activity, vigorous physical activity	No significant differences found between the experimental and control groups for any outcome variables (all *p* > 0.975).

*β* beta coefficient, *CI* confidence interval, *K* Kindergarten, ages 5–6 years in USA, *m* metres, *METs* metabolic equivalents, *MVPA* moderate-to-vigorous physical activity, *n* number, *OR* odds ratio, *UK* United Kingdom, *USA* United States of America, *Z* Wilcoxon Signed Ranks test statistic

### Quality assessment

Study quality assessment results are provided in Table 2. Using the EPHPP criteria, only one study was rated as strong (i.e., having no components with a weak rating), and a majority (75%) were rated as weak. No studies rated as moderate or strong assessed walking or cycling separately from each other. Selection bias was the quality component most frequently rated as weak. In many cases this was a consequence of undertaking direct observation of park users, where no measures of representativeness could be ascertained. In general, studies lacked robust means of ensuring generalisability. The use of direct observation made it difficult to fully control for confounders. Relevant information on traffic and street type was also frequently missing in studies that assessed the effectiveness of streetscape interventions. Of the studies where it was appropriate (i.e., where there was a control/comparison group or site), 28% reported on consistency between treatment conditions. Five studies noted distance between treatment sites, but no studies measured contamination directly. Cost of the intervention was reported in seven studies, all of which were conducted in the US. These ranged from US$45,000 per park for implementation of fitness zones to US$5.5 million for two major playfield renovations.

**Table 2:** Quality assessment of studies included in review

Author; year	Selection bias	Study design	Confounders	Blinding	Data collection	Withdrawals	Global score
Beenackers; 2012	Weak	Moderate	Strong	Moderate	Weak	Weak	Weak
Boarnet; 2005	Weak	Moderate	Weak	Moderate	Moderate	N/A	Weak
Brown; 2008	Weak	Moderate	Strong	Moderate	Strong	Weak	Weak
Clark; 2014	Weak	Moderate	Weak	Moderate	Strong	N/A	Weak
Cohen; 2012	Weak	Moderate	Moderate	Moderate	Strong	N/A	Moderate
Cohen; 2014	Weak	Moderate	Weak	Moderate	Strong	N/A	Weak
Cohen; 2015	Weak	Moderate	Weak	Moderate	Strong	N/A	Weak
D’Haese; 2015	Moderate	Moderate	Strong	Weak	Strong	Moderate	Moderate
Dill; 2014	Weak	Moderate	Strong	Moderate	Strong	Moderate	Moderate
Fitzhugh; 2010	Weak	Moderate	Weak	Moderate	Weak	N/A	Weak
Giles-Corti; 2013	Weak	Moderate	Strong	Moderate	Weak	Moderate	Weak
Goodman; 2014	Weak	Moderate	Strong	Moderate	Strong	Weak	Weak
Gustat; 2012	Moderate	Moderate	Moderate	Moderate	Weak	N/A	Moderate
Harduar-Morano; 2008	Strong	Weak	Weak	Weak	Weak	N/A	Weak
Knuiman; 2014	Weak	Moderate	Strong	Moderate	Weak	Weak	Weak
Lott; 1979	Weak	Moderate	Weak	Moderate	Weak	N/A	Weak
McDonald; 2013	Moderate	Moderate	Strong	Moderate	Moderate	N/A	Strong
Morrison; 2004	Weak	Weak	Weak	Moderate	Weak	Moderate	Weak
Parker; 2011	Weak	Moderate	Weak	Moderate	Weak	N/A	Weak
Parker; 2013	Weak	Moderate	Weak	Moderate	Weak	N/A	Weak
Quigg; 2011	Weak	Moderate	Strong	Moderate	Strong	Strong	Moderate
Ranchod; 2013	Weak	Moderate	Moderate	Moderate	Strong	Strong	Moderate
Roemmich; 2014	Weak	Moderate	Weak	Moderate	Strong	N/A	Weak
Shu; 2014	Weak	Moderate	Weak	Moderate	Weak	N/A	Weak
Tester; 2009	Weak	Moderate	Weak	Moderate	Strong	N/A	Weak
Veitch; 2012	Weak	Moderate	Weak	Moderate	Strong	N/A	Weak
West; 2011	Weak	Moderate	Weak	Moderate	Weak	Weak	Weak
West; 2015	Weak	Moderate	Moderate	Moderate	Weak	Moderate	Weak

### Differential effects by ethnicity or socio-economic status

Four studies investigated whether built environment effects differed by ethnicity/race or SES, using a range of different approaches. The iConnect CBA study of new walking and cycling routes in three UK municipalities over two years found no significant interaction between the intervention and education, income or employment when using walking and cycling as the outcome [56]. When examining use of the infrastructure, lower educational level and income, but not ethnicity, were associated with less use. Compared with an annual income of >£40,000, an income of <£20,000 was associated with a 23% lower likelihood of infrastructure use. Having less than tertiary-level educational level, compared with tertiary education, was associated with a 10–20% lower likelihood of infrastructure use, though the effect was only statistically significant at one-year (not two-year) follow-up. People who were not working, who were retired or were students (compared with working people) were less likely to use the infrastructure at two-year follow-up. Students were particularly unlikely to use the infrastructure (relative ratio = 0.20) [35].

A randomized controlled trial of the effects of Los Angeles park improvements informed by community engagement found a significant increase in the number of white park users, but no significant changes for black, Hispanic or other users [57]. Installation of a one-mile bike lane in New Orleans was associated with increases in mean daily cycling counts on the intervention street for both whites (from 48.9 to 192.9/day) and blacks (from 22.8 to 61.3/day; results for other racial/ethnic groups not reported), while cycle counts on adjacent control streets dropped post-intervention. The interaction of intervention, time and race/ethnicity was not statistically significant [58]. In a longitudinal study across several US cities, the effect of change in neighbourhood recreational facility density on change in recreational physical activity (mean follow-up 3.2 years) did not differ significantly by income or race/ethnicity [59].

### Impact of built environment on physical activity, active transport, or visitation/use of settings

Table 3 summarises the key findings in relation to the impact of the built environment on physical activity, active transport, and visits or use of settings. With the exception of two studies showing no significant impact [60, 61], and one finding a negative impact [62], all others reported a significant positive impact on the outcomes of interest. For those studies rated higher quality (i.e., moderate or strong ranking using the EPHPP criteria), significant positive impacts were found for the following:

- Interventions involving multiple streetscape improvements on active transport in children, and on physical activity in adults [63, 64]
- Installation of park or playground equipment and active transport and physical activity in adults [57, 63]
- Multiple component park renovations on children’s physical activity (for children of lower body size only) [65]
- Temporary road closures and provision of play equipment on children’s physical activity [66]
- Increased density of recreation facilities in the neighbourhood on physical activity in adults [59]

**Table 3:** Key findings and strength of evidence^a^ for impact of built environment on physical activity, active transport, and visitation or use of settings

Intervention mechanism	Intervention medium	Active transport (total walking, total cycling, walking or cycling for transport)			Physical activity (total physical activity, moderate physical activity, moderate-to-vigorous physical activity, recreational physical activity)			Visitation/use of settings		
		Adults	Children	All ages	Adults	Children	All ages	Adults	Children	All ages
Active transport infrastructure	Bicycle boulevard/bike lane installation	↓[62]			~ [62]			↑↑[58, 91]		↑[93]
	Multiple streetscape components for walking or cycling (including two or more of: crosswalk and sidewalk improvements, improved and covered bike parking, installation of traffic calming features (raised platforms, zebra crossings) and parking bays; creating safe places to walk)	↑[92] **~** ^**+**^ [63]	**↑** ^**++**^ [64] ↑[85]		↑↑[90, 92] **↑** ^**+**^ [63]					↑↑[92, 95]
	New greenways		~[90]^b^	↑[89]	↑[98] ~ [60]		↑[89]			
	Traffic free bridges and boardwalks	↑[56]			↑[56]					
	Wayfinding and distance signage on community trails									~ [61]
Parks and playgrounds	Installation of fitness equipment/playground equipment	**↑** ^**+**^ [57] ~+ [63]			**↑** ^**+**^ **↑** ^**+**^ [57, 63]					
	Multiple component park renovations (including two or more of: new equipment, walking track, fencing, landscaping, surfaces, lights)				↑[90]	**~** ^**+**c^ [65]	↑↑↑[86, 96, 97] ~ [88]			↑↑↑[88, 96, 97]
	Removal of seating				↑[94]	~ [94]				↓[94]
	Retrofiting existing green space into pocket parks				↑[87]			↑[87]		
	Temporary road closures and play equipment					↑^**+**^ [66]				
Walkability components	Access to/availability of public transit	↑[71]			↑[86]					
	Destination accessibility	↑↑[69, 71]			↑[69]					
	Land-use mix	↑[71]								
	Recreation facility density				↑^**+**^ [59]					
	Residential density	↑[70] ~ [71]								
	Street connectivity	↑[71]			↑[70]					

^a^Strength of evidence determined by quality assessment rating of each study using a modified version of the *Evaluation of Public Health Practice Projects Quality Assessment Tool* (EPHPP) as described in the supplementary information (Additional File 3)

^b^Effect for school transport mode only

^c^Moderating effect of body size, whereby intervention was effective in increasing activity in children of lower body size, and decreased activity in children of higher body size

^+^Moderate evidence

^++^Strong evidence

~ Inconsistent results or no impact of built environment on physical activity behaviour(s) or visitation/use of setting(s)

↓ Negative impact of built environment on physical activity behaviour(s) or visitation/use of setting(s); weak evidence

↑ Positive impact of built environment on physical activity behaviour(s) or visitation/use of setting(s); weak evidence

## Discussion

The aims of this systematic review were to provide an update on the evidence for the impact of the built environment on physical activity behaviours, and to systematically explore the effectiveness of these interventions by ethnicity and SES. Drawing from best practice, we took a systematic approach to identifying and screening literature, data extraction, and quality assessment of relevant literature.

Findings showed a consistent positive effect of walkability components, provision of quality parks and playgrounds, and installation of or improvements in active transport infrastructure on active transport, physical activity, and visits or use of settings. Impacts on activity dimensions were observed in line with expectations (e.g., walkability components were related to active transport) [14]. Additionally, some interesting patterns emerged whereby improvements in non-specific activity dimensions occurred, such as installation of fitness or playground equipment increasing active transport to that setting, and improving the streetscape for walking and cycling increasing physical activity behaviours beyond active transport. Positive impacts were observed for children and adults alike, although there was a paucity of research including older adults. Taken together, these findings provide new evidence for the substantial promise that built environment interventions hold for improving physical activity behaviours across a population and are in keeping with previous systematic reviews [12–27]. There remains a substantial gap in understanding relationships with cycling behaviours. None of the studies rated as strong or moderate quality assessed cycling independently of other active transport modes.

Most analyses found no statistically significant differences in intervention effect by ethnicity or SES. However, one study found that new walking and cycling routes were used more by people with higher incomes, higher educational level and by people who were currently employed. Another study of park improvements found increased number of white users, but no significant change for black, Hispanic or other park users. Although these two studies provide a limited basis from which to draw inferences, the possibility that infrastructure improvements may predominantly benefit socioeconomically advantaged groups warrants further exploration in future evaluations.

As identified in previous reviews, there was substantial variability in the methodology, measures, and analytical processes used to evaluate the impact of environmental interventions. Overall, this made comparisons between studies and intervention delivery approaches difficult. In part, this variability may be a by-product of the significant differences in intervention typologies and associated research designs. There remains, however, a need for improved study quality, which may mitigate this somewhat (e.g., consistent application of objective assessment of physical activity, including appropriate control conditions, employment of strategies to improve response rates, adequate consideration of confounders, etc.). Our quality assessment rated three-quarters of included studies as weak. Studies were frequently limited by lack of representativeness and generalisability.

In most cases, blinding was not described in studies. Following the scoring protocol of the EPHPP, this earned a “Moderate” rating. It is worth noting there are ethical issues to be considered with participant blinding (such as informed consent), and that these can sometimes conflict with quality assessment criteria, where blinding is desirable to reduce bias associated with participant self-report or reactivity [67]. We chose to retain this category for consistency with the EPHPP and previous research that has used this tool. An indepth consideration of the issues surrounding the evaluation of blinding in quality assessment is beyond the scope of this review, but should be taken into account in future reviews in this field.

Few studies comprehensively controlled for confounders. While sex and age were commonly controlled or stratified for in analyses, only some included robust measures of SES or ethnicity. Depending on study design, area-level SES (and ethnicity characteristics) may suffice in order to mitigate challenges around collecting these data at the individual level. In their systematic review, Schule et al. [25] recommended the use of comparable characteristics of neighbourhood-level SES in studies of the built environment, recognising the need to “disentangle health impacts and identify vulnerable neighbourhoods and population groups”.

Issues around contamination and consistency across intervention sites were rarely considered and were not controlled for in any analyses. Future studies would also benefit from considering neighbourhood self-selection as a possible confounder. In their systematic review, McCormack et al. [12] reported “an attenuation in associations between built environment characteristics and physical activity after accounting for neighbourhood self-selection”. Likewise, baseline activity was rarely controlled for in analyses. One recent study to consider this factor revealed that existing cycling behaviour, as well as distance to the intervention site, was associated with increased use of a new bike path in Australian adults [68].

Overall, there was a lack of research specific to children or older adults. It is possible that the adult-centric approach to characterising built environments may not be sufficiently capturing environments that matter to younger populations. For example, in their earlier review, Sugiyama et al. [29] found no link between route safety and traffic features and adults’ utilitarian and recreational walking, while these factors have previously been linked with children’s active transport [32]. A recent systematic review showed that where youth physical activity was geo-located (i.e., with global positioning systems devices and geographic information systems), greater proportions of activity took place in streets and urban venues than in green spaces [15].

In keeping with Hunter et al. [13], reporting of intervention costs was uncommon in the studies included in this review. The lack of clear and consistent reporting of intervention cost limited any ability to determine whether a relationship existed between expenditure and behaviour change, and whether costs differed significantly between intervention types. This may also lead to bias in studies of benefit:cost analyses for built environment interventions because only a small portion of evidence is presented, and it is not clear why some studies report costs and other don’t [24, 27]. Moreover, the low quality of evidence found overall negatively impacts confidence in estimations of cost-effectiveness of built environment interventions [41]. Given that the costs of included interventions varied widely (ranging from USD45,000 to USD5.5 million in the current study), it is essential that clear reporting of intervention costs occurs, and that intervention studies also include a cost-benefit component.

Compared with infrastructural interventions, relocation studies are less hindered by infrastructural cost. RESIDE is perhaps the first study of its kind – a natural experiment involving longitudinal data collection with new residents of new housing developments [69]. The study commenced in 2003 prior to participants moving, with follow-up data collection occurring with participants approximately 12 months after their baseline (pre-moving) survey. Improvements in walkability (specifically, objectively-assessed destination accessibility, residential density, street connectivity, and access to/availability of public transit) were associated with increases in physical activity and use of active transport [69–71]. This important study demonstrates the value of well-designed residential relocation studies.

The substantial inconsistency in timing and number of follow-up measures post exposure (i.e., through intervention or moving) limits a clear understanding of the impact of the built environment on physical activity. In their systematic review of natural or quasi-experiments, Mayne et al. [14] found that stronger impacts on physical activity were found where studies included longer follow-up times. This phenomenon was observed in the iConnect study, in which no effect on behaviour was observed one year after a major environmental intervention, but significant changes were found at the two-year follow-up [56]. Pragmatic and economic factors may limit the feasibility of repeated and longer-term follow-up periods in this research field, but where possible this should be encouraged.

Finally, it is possible that publication bias affected the balance of studies included in this review. In general, registration or public release in other forms of study protocols prior to the publication of findings provides a check on selective reporting of study results.

### Strengths and limitations

This systematic review focused on physical activity behaviours and use or visitation of settings, and did not include more distal health measures such as body size [72]. While these outcomes are unquestionably important, this approach was taken recognising that: (a) a substantial period of time exists between sustained activity behaviours and manifestation of associated health outcomes, and (b) studies in this field rarely examine effects of the built environment on activity behaviours for a sufficient period of time to expect meaningful changes in health outcomes that are downstream from improved physical activity behaviours.

The broad inclusion criteria used here are a strength, drawing on studies across all quality assessment categories as well as considering findings in light of study quality. However, the exclusion of grey literature could have been a limiting factor, in that it is possible that significant new lines of inquiry might have been overlooked.

While acknowledging the important role of perceptions on the relationship between the built environment and activity behaviours [73, 74], this review focused only on objectively assessed built environment features. The association between the built environment and activity varies, depending on whether perceived or objective measures are used [73, 75–77]. Moreover these differences are not consistent, and vary along with individual factors such as education level, income, body size, and physical activity [74, 78]. Individuals who are more physically active may be more familiar with their local environment, and more aware of both positive and negative features, than those who are less active [79]. In the interest of providing clear and direct information to inform policy and practice that was not complicated or hindered by individual perceptions, we chose to focus on objective measures of the environment. Future reviews may consider replicating the current study with a focus on perceived environmental features only, while others may undertake the task of considering both objective and subjectively-assessed environments. There is an emerging body of research that simultaneously considers both objective and subjective measures of the environment in relation to physical activity [76]; offering challenges and opportunities for future reviews in this area.

Our focus was on being able to isolate the effect of specific built environment features or interventions, thus studies that employed aggregate measures (e.g., neighbourhood walkbability) were excluded from this review. A range of robust studies exist in the field that have utilized aggregate measures [80, 81]. The exclusion of this research limits an understanding of the combination of features that encompass a pedestrian or cyclist friendly environment. However, this approach was taken in the interest of generating specific findings that could be translated into policy and practice. Measures that combine variables may be difficult to interpret, if it is not possible to determine which components are most effective. Future reviews with differing aims could consider including aggregate scores, and might extend this to broader aggregate concepts such as obesogenic environments [82].

There have been calls for more systematic approaches in reviews in this field [83]. This study employed robust systematic procedures following best practice, and improved on existing reviews by conducting quality assessment of articles, considering article quality when summarising results, including literature for children and adults, excluding cross-sectional studies, and attempting to isolate the effect of the built environment from other interventions occurring in studies. With the intent of providing evidence from the most robust evidence possible and undertaking a process that was as replicable as possible, as well as drawing from existing high quality reviews in this field [12, 14, 16, 30, 31, 46], this review did not include grey literature or involve forwards citation searches. Future reviews may undertake these processes to capture an evidence base that is as broad and up-to-date as possible [45, 84]. Although our global quality assessment score does not include measures of study contamination, consistency, health equity, or intervention cost, these factors were systematically considered and presented in the context of understanding the overall quality of studies presented in this review.

## Conclusion

The systematic and comprehensive approach to examining study quality and contextualising findings in light of study quality in this review adds strength to the evidence base for the positive impact of built environments on physical activity behaviours, active transport, and visits to/use of activity settings. Improving neighbourhood walkability, quality of parks and playgrounds, and providing adequate active transport infrastructure is likely to generate positive impacts on activity in children and adults. Specifically, findings from the current review suggest that the following show promise for increasing active transport and physical activity levels in children and adults: multiple streetscape components for walking or cycling (including two or more of: crosswalk and sidewalk improvements, improved and covered bike parking, installation of traffic calming features (raised platforms, zebra crossings) and parking bays; creating safe places to walk); bike boulevard/lane installation; new greenways; traffic free bridges and boardwalks; installation of fitness/playground equipment; multiple park renovations (including two or more of: new equipment, walking tracks, fencing, landscaping, surfaces, lights); removal of park/playground seating; retrofitting existing spaces into pocket parks; temporary road closures and play equipment; access to and availability of public transport; higher residential, destination, and recreation density; increased street connectivity; and increased land use mix. The strongest evidence existed for multiple streetscape components (adult PA, child AT), installation of fitness equipment (adult PA and AT), temporary road closures and play equipment (child PA), and recreation facility density (adult PA).

Numerous limitations in the evidence base exist. In particular, the possibility that the benefits of infrastructure improvements may be inequitably distributed requires further investigation. Notwithstanding the significant challenges in terms of research design, many opportunities to improve the quality of evidence are clear, including strategies to improve response rates and representativeness, use of valid and reliable measurement tools, cost-benefit analyses, and adequate controlling for confounders.

## Additional files

Additional file 1: Search terms. (DOCX 11 kb)

Additional file 2: Data extraction form. (DOCX 13 kb)

Additional file 3: Adaptations made to the Evaluation of Public Health Practice Projects Quality Assessment Tool (EPHPP) scoring criteria. (DOCX 25 kb)

Additional file 4: Effective Public Health Practice Project (EPHPP) Quality Assessment Tool for Quantitative Studies - Component Ratings of Study. (DOCX 12 kb)

## Abbreviations

EPHPP: Evaluation of Public Health Practice Projects Quality Assessment Tool
SES: Socioeconomic status
UK: United Kingdom
US: United States
USD: United States dollars
