# Supplementary material for: Systematic literature review of built environment effects on physical activity and active transport – an update and new findings on health equity
Source: Int J Behav Nutr Phys Act. 2017 Nov 16;14:158. doi: 10.1186/s12966-017-0613-9 (PMC5693449; doi:10.1186/s12966-017-0613-9)
Supplement: Supplementary file 1 — Search terms. (DOCX 11 kb) [file 12966_2017_613_MOESM1_ESM.docx]

### Additional File 1

#### Search terms

AB,TI("active transport*" OR walk* OR cyclist* OR bik* OR bicycl* OR cyclist OR cycling OR "active travel*" OR commute* OR "physical activ*" OR "physically active" OR "transport mode" OR "transportation mode" OR "travel mode" OR pedestrian* OR "traffic volume" OR "traffic count")

AND AB,TI(streetscape OR "self-explaining road*" OR "self explaining road*" OR neighbourhood OR neighborhood OR "physical environment*" OR "urban environment*" OR "suburban environment*" OR "built environment*" OR "community environment*" OR "travel environment*" OR "street environment*" OR "road environment*" OR "shared space*" OR "traffic calm*" OR "smart growth" OR "new urbanism" OR "urban form" OR "urban design" OR walkability OR "residential density" OR "community design" OR "city planning" OR "environment design" OR "urban renewal" OR sidewalk OR footpath OR "green space*" OR "recreational facilit*" OR "public transit" OR "public transport*" OR "block size*" OR "street connectivity" OR "open space*" OR woonerf OR "naked street*" OR "sustainable safety" OR (cycl* AND trail*) OR (bik* AND trail*) OR (bicycle* AND trail*) OR (walk* AND trail*) OR (pedestrian* AND trail*) OR infrastructure OR "community-based" OR (route AND school) OR (bik* AND (path* OR lane*)) OR (bicycle* AND (path* OR lane)) OR (cycl* AND (path* OR lane)) OR cycleway)

AND AB,TI(intervention* OR longitudinal OR follow* OR chang* OR effect* OR initiative* OR experiment* OR evaluate* OR evidence OR impact* OR "before-after" OR "time-series" OR program* OR "prospective stud*")
