# Supplementary material for: Systematic literature review of built environment effects on physical activity and active transport – an update and new findings on health equity
Source: Int J Behav Nutr Phys Act. 2017 Nov 16;14:158. doi: 10.1186/s12966-017-0613-9 (PMC5693449; doi:10.1186/s12966-017-0613-9)
Supplement: Supplementary file 2 — Data extraction form. (DOCX 13 kb) [file 12966_2017_613_MOESM2_ESM.docx]

### Additional File 2

#### Data extraction form

| **General information** | Date form completed |  |
| --- | --- | --- |
|  | Initials of person extracting the data |  |
|  | Study ID |  |
|  | IDs of other reports of this study (e.g., duplicate publications, follow-up studies) |  |
|  | Article title |  |
|  | Lead author |  |
|  | Year of publication |  |
|  | Funding source |  |
|  | Possible conflicts of interest |  |
|  | Notes |  |
| Population and setting | Population description |  |
|  | Setting |  |
|  | Inclusion criteria |  |
|  | Exclusion criteria |  |
|  | Method/s of recruitment of participants |  |
|  | Notes |  |
| Methods | Aim of study |  |
|  | Design (e.g., parallel, crossover, non-RCT) |  |
|  | Unit of allocation |  |
|  | Start date |  |
|  | End date |  |
|  | Duration of participation (from recruitment to last follow-up) |  |
|  | Notes |  |
| Participants | Total number randomised (or total population at start of study for non randomised controlled trials) |  |
|  | Clusters (if applicable, number, type, number of people per cluster) |  |
|  | Withdrawals and exclusions |  |
|  | Age (years) |  |
|  | Sex (% male) |  |
|  | Race/ethnicity |  |
|  | Other relevant socio-demographics |  |
| Intervention groups | Number randomised to each group (specify whether number of people or clusters) |  |
|  | Description of groups (e.g., content, dose, components) |  |
|  | Control group (yes/no) |  |
|  | Duration of treatment period |  |
|  | Timing (e.g., frequency, duration of each episode) |  |
|  | Delivery (e.g., mechanism, medium) |  |
|  | Economic variables (i.e., intervention cost, changes in other costs as result of intervention) |  |
|  | Notes on consistency |  |
| Outcomes | Outcome name(s) |  |
|  | Instrument(s) |  |
|  | Time points measured |  |
|  | Validity and reliability of instrument(s) |  |
|  | Imputation of missing data (e.g., assumptions made for intention-to-treat analysis) |  |
|  | Notes |  |
| Results | Key findings |  |
|  | Adjusted for confounders |  |
|  | Differences found were statistically significant (yes/no) |  |
