# Supplementary material for: Systematic literature review of built environment effects on physical activity and active transport – an update and new findings on health equity
Source: Int J Behav Nutr Phys Act. 2017 Nov 16;14:158. doi: 10.1186/s12966-017-0613-9 (PMC5693449; doi:10.1186/s12966-017-0613-9)
Supplement: Supplementary file 3 — Adaptations made to the Evaluation of Public Health Practice Projects Quality Assessment Tool (EPHPP) scoring criteria. (DOCX 25 kb) [file 12966_2017_613_MOESM3_ESM.docx]

### Additional File 3

#### Adaptations made to the Evaluation of Public Health Practice Projects Quality Assessment Tool (EPHPP) scoring criteria

| **Quality assessment categories** | **Original question and responses** | **Chillon adaptation** | ***Study adaptation from Chillon*** |
| --- | --- | --- | --- |
| **A Selection Bias** | 1. Are the individuals selected to participate in the study likely to be representative of the target population? | Adapted to the school level using: “Are the schools selected to participate in the study likely to be representative of the target population?” | *Modified to integrate both previous versions: "Are the individuals/schools selected to participate in the study likely to be representative of the target population?"* |
|  | Very Likely | n/c | *n/c* |
|  | Somewhat Likely | n/c | *n/c* |
|  | Not Likely | n/c | *n/c* |
|  | Can’t tell | n/c | *Clarified by including the text: (also used when direct observation is the method of measurement)* |
|  | 2. What percentage of selected individuals agreed to participate? | Adapted to the school level using: “What percentage of selected schools agreed to participate?” If school level data was not reported or if the study had only individual level data, we reported on the individuals that reported the main outcome (active transportation to school). If both parents and children reported the main outcome, then parent’s data were used. | *Modified to integrate both previous versions: "What percentage of selected individuals/schools agreed to participate?"* |
|  | 80 - 100% Agreement | n/c | *n/c* |
|  | 60 - 79% Agreement | n/c | *n/c* |
|  | Less than 60% Agreement | n/c | *n/c* |
|  | Not Applicable | n/c | *Clarified by including the text: (also used when direct observation is the method of measurement)* |
|  | Can’t tell | n/c | *n/c* |
|  | 3. Rate this section (see dictionary) | n/c | *n/c* |
|  | Strong | n/c | *n/c* |
|  | Moderate | n/c | *n/c* |
|  | Weak | n/c | *n/c* |
| **B Allocation Bias** | 4. Indicate the study design | n/c | *n/c* |
|  | Randomized controlled trial | The design of a group randomized controlled trial was included in the first category, in the same level as randomized controlled trial studies. | *n/c* |
|  | Controlled Clinical Trial | n/c | *n/c* |
|  | Cohort analytic (two group pre and post) | The third category was modified from “cohort analytic (two group pre + post)” to a “two group pre + post design”. | *Clarified to include both as follows: "Cohort analytic (two groups pre & post)/two group pre+post design"* |
|  | Case control study | n/c | *n/c* |
|  | Cohort (one group pre + post (before and after) | The fifth category was modified from “cohort (one group pre + post (before and after))” to a “one group pre + post (before and after) design”. | *Clarified to include both as follows: "Cohort (one group pre + post (before and after))/one group pre + post (before and after) design"* |
|  | Interrupted time series | n/c | *Changed to include uncontrolled cross sectional pretest-post test: "Interrupted Time Series/uncontrolled cross sectional pretest posttest"* |
|  |  |  | *7. Other category added* |
|  | 5. Is the method of random allocation stated? | n/c |  |
|  | Yes | n/c | *n/c* |
|  | No | n/c | *n/c* |
|  |  |  | *3. Not applicable category added* |
|  | 6. If the method of random allocation is stated, is it appropriate? | n/c | *n/c* |
|  | Yes | n/c | *n/c* |
|  | No | n/c | *n/c* |
|  |  |  | *3. Not applicable category added* |
|  | 7. Was the method of random allocation reported as concealed? | n/c | *n/c* |
|  | Yes | n/c | *n/c* |
|  | No | n/c | *n/c* |
|  |  |  | *3. Not applicable category added* |
|  | 8. Rate this section (see dictionary) |  | *n/c* |
|  | Strong | n/c | *n/c* |
|  | Moderate | n/c | *n/c* |
|  | Weak | n/c | *n/c* |
| **C Confounders** | 9. Prior to the intervention were there between group differences for important confounders reported in the paper? | n/c | *n/c* |
|  | Yes | n/c | *n/c* |
|  | No | n/c | *n/c* |
|  | Can't tell | n/c | *Clarified as follows: "Can't tell (e.g., data presented but no evidence of testing for significant differences between variables presented)"* |
|  |  | If the study did not have a control group, the assessment was "Not applicable" | *Clarified as follows: "N/A (no control or comparison group; n = 1 site/setting)"* |
|  | 10. Relevant confounders reported in the study. | n/c | *All articles were systematically screened for consideration of common confounders as follows: age, sex, ethnicity, socio-economic status, marital status, education, health status, and pre-intervention score. Scoring was as follows: 1. Controlled or stratified for confounder in analyses; 2. Did not control or stratify for confounder based on preliminary analyses (tested for statistical significance between groups at baseline but none found); 3. Did not control or stratify for confounder, no justification provided or can't tell - no evidence of testing for significance observed or variable not reported. Additionally, study-specific confounders (e.g., traffic) were identified and noted in a free-text box.* |
|  | 11. If there were differences between groups for important confounders, were they adequately managed in the analysis? | n/c | *n/c* |
|  | Yes | n/c | *n/c* |
|  | No | n/c | *n/c* |
|  | Not Applicable | n/c | *Clarified to include the text "including when response to option 9 is "3, Can't Tell""* |
|  | 12. Were there important confounders not reported in the paper? |  | *Clarified that these were aside from the variables already considered in Q10* |
|  | Yes | n/c | *n/c* |
|  | No | n/c | *n/c* |
|  | 13. Relevant confounders NOT reported in the study (free-text) | n/c | *n/c* |
|  | 14. Rate this section (see dictionary) |  |  |
|  | Strong | n/c | *n/c* |
|  | Moderate | n/c | *n/c* |
|  | Weak | n/c | *n/c* |
| **D Blinding** | 15. Was (were) the outcomes assessor(s) blinded to the intervention or exposure status of participants? | n/c | *n/c* |
|  | Yes | n/c | *n/c* |
|  | No | n/c | *n/c* |
|  | Can't tell | If the questionnaire was self-administered, the answer was 3 (Can’t tell). | *n/c* |
|  |  |  | *Added "Not applicable" category* |
|  | 15a. Was (were) participants aware of the research question? | n/c | *n/c* |
|  | Yes | n/c | *n/c* |
|  | No | n/c | *n/c* |
|  | Can't tell | If the questionnaire was self-administered, the answer was 3 (Can’t tell). | *n/c* |
|  |  |  | *Added "Not applicable" category* |
|  | 16. Rate this section (see dictionary) | n/c | *n/c* |
|  | Strong | n/c | *n/c* |
|  | Moderate | n/c | *n/c* |
|  | Weak | n/c | *n/c* |
|  | Not Applicable | n/c | *n/c* |
| **E Data Collection Methods** | 17. Were data collection tools shown or are they known to be valid? | n/c | *n/c* |
|  | Yes | n/c | *n/c* |
|  | No | If there was no reference to the data collection tools used in the study or no indication about validity or reliability, then it was assumed to not be available and the answer was "No" | *n/c* |
|  | 18. Were data collection tools shown or are they known to be reliable? | n/c | *n/c* |
|  | Yes | n/c | *n/c* |
|  | No | If there was no reference to the data collection tools used in the study or no indication about validity or reliability, then it was assumed to not be available and the answer was "No" | *n/c* |
|  | 19. Rate this section (see dictionary) | n/c | *n/c* |
|  | Strong | n/c | *n/c* |
|  | Moderate | n/c | *n/c* |
|  | Weak | n/c | *n/c* |
| **F Withdrawals and Drop-outs** | 20. Indicate the percentage of participants completing the study. (If the percentage differs by groups, record the lowest) | |  |
|  | 80 - 100% |  |  |
|  | 60 - 79% |  |  |
|  | Less than 60% |  |  |
|  | Not Reported |  |  |
|  | Not Applicable | If the study had only one measure (pre or post), the assessment was "Not applicable" | *Clarification expanded to include other study types and methods: If the study had only one measure (pre or post) OR if direct observation of a setting OR if a repeat cross-sectional study the assessment was "Not applicable"* |
|  | 21. Rate this section (see dictionary) | n/c | *n/c* |
|  | Strong | n/c | *n/c* |
|  | Moderate | n/c | *n/c* |
|  | Weak | n/c | *n/c* |
|  | Not Applicable | n/c | *n/c* |
| **G Analysis** | 22. Is there a sample size calculation or power calculation? | n/c | *n/c* |
|  | Yes | n/c | *n/c* |
|  | Partially | n/c | *n/c* |
|  | No | n/c | *n/c* |
|  | 23. Is there a statistically significant difference between groups? | n/c | *Question clarified to read: 23. Is there a statistically significant difference in key variables between groups (if uncontrolled) or between time points (if cross sectional)?* |
|  | Yes | n/c | *n/c* |
|  | No | n/c | *n/c* |
|  | Not Reported | n/c | *n/c* |
|  | 24. Are the statistical methods appropriate? | n/c | *n/c* |
|  | Yes | n/c | *n/c* |
|  | No | n/c | *n/c* |
|  | Not Reported | When statistical analyses were not provided, this question was answered by reviewing the results for each study | *n/c* |
|  | 25. Indicate the unit of allocation. | n/c | *n/c* |
|  | Community | n/c | *n/c* |
|  | Organization/Institution Group | The unit of analysis in was organization/institution when results were organized for each school | *n/c* |
|  | Provider | n/c | *n/c* |
|  | Individual | The unit of analysis in was individual when results were organized without separating schools | *n/c* |
|  | 26. Indicate the unit of analysis. | n/c | *n/c* |
|  | Community | n/c | *n/c* |
|  | Organization/Institution Group | The unit of analysis in was organization/institution when results were organized for each school | *n/c* |
|  | Provider | n/c | *n/c* |
|  | Individual | The unit of analysis in was individual when results were organized without separating schools | *n/c* |
|  | 27. If the unit of allocation and the unit of analysis are different, was the cluster analysis done? | n/c | *n/c* |
|  | Yes | n/c | *n/c* |
|  | No | n/c | *n/c* |
|  | Not Applicable | n/c | *n/c* |
|  | 28. Is the analysis performed by intervention allocation status (i.e. intention to treat) rather than the actual intervention received? | n/c | *n/c* |
|  | Yes | n/c | *n/c* |
|  | No | n/c | *n/c* |
|  | Can't tell | n/c | *n/c* |
|  | Not Applicable | n/c | *n/c* |
|  | 29. Comments (free-text) | n/c | *n/c* |
| **H Intervention Integrity** | 30. What percentage of participants received the allocated intervention or exposure of interest? | n/c | *n/c* |
|  | 80 - 100% | 80 - 100% | *n/c* |
|  | 60 - 79% | 60 - 79% | *n/c* |
|  | Less than 60% | Less than 60% | *n/c* |
|  | Not Reported | Not Reported | *n/c* |
|  | Not Applicable | Not Applicable | *n/c* |
|  | 31. Was the consistency of the intervention measured? | n/c | *n/c* |
|  | Yes | n/c | *n/c* |
|  | No | If there was no indication about consistency in the study, it was assumed to not be reported or available and the answer was "No" | *The original coding criteria of the EPHPP was employed, rather than the adaptation from Chillon here* |
|  | Not Reported | If there was no indication about consistency in the study, it was assumed to not be reported or available and the answer was "No" | *n/c* |
|  | Not Applicable | n/c | *n/c* |
