# Supplementary material for: Systematic literature review of built environment effects on physical activity and active transport – an update and new findings on health equity
Source: Int J Behav Nutr Phys Act. 2017 Nov 16;14:158. doi: 10.1186/s12966-017-0613-9 (PMC5693449; doi:10.1186/s12966-017-0613-9)
Supplement: Supplementary file 4 — Effective Public Health Practice Project (EPHPP) Quality Assessment Tool for Quantitative Studies - Component Ratings of Study. (DOCX 12 kb) [file 12966_2017_613_MOESM4_ESM.docx]

### Additional File 4

**Effective Public Health Practice Project (EPHPP) Quality Assessment Tool for Quantitative Studies - Component Ratings of Study**

For each of the six components A – F, use the following descriptions as a roadmap.

**A)  SELECTION BIAS**

**Strong:** The selected individuals are very likely to be representative of the target population (Q1 is 1) **and** there is greater than 80% participation (Q2 is 1).

**Moderate:** The selected individuals are at least somewhat likely to be representative of the target population (Q1 is 1 or 2); **and** there is 60 - 79% participation (Q2 is 2). ‘Moderate’ may also be assigned if Q1 is 1 or 2 and Q2 is 5 (can’t tell).

**Weak:** The selected individuals are not likely to be representative of the target population (Q1 is 3); **or** there is less than 60% participation (Q2 is 3) **or** selection is not described (Q1 is 4); and the level of participation is not described (Q2 is 5).

**B)  DESIGN**

**Strong:** Will be assigned to those articles that described RCTs and CCTs.

**Moderate:** Will be assigned to those that described a cohort analytic study, a case control study, a cohort design, or an interrupted time series.

**Weak:** Will be assigned to those that used any other method or did not state the method used.

**C)  CONFOUNDERS**

**Strong:** Will be assigned to those articles that controlled for at least 80% of relevant confounders (Q1 is 2); **or** (Q2 is 1).

**Moderate:** Will be given to those studies that controlled for 60–79% of relevant confounders (Q1 is 1) **and** (Q2 is 2).

**Weak:** Will be assigned when less than 60% of relevant confounders were controlled (Q1 is 1) **and** (Q2 is 3) **or** control of confounders was not described (Q1 is 3) **and** (Q2 is 4).

**D)  BLINDING**

**Strong:** The outcome assessor is not aware of the intervention status of participants (Q1 is 2); **and t**he study participants are not aware of the research question (Q2 is 2).

**Moderate:** The outcome assessor is not aware of the intervention status of participants (Q1 is 2); **or** the study participants are not aware of the research question (Q2 is 2); **or b**linding is not described (Q1 is 3 and Q2 is 3).

**Weak:** The outcome assessor is aware of the intervention status of participants (Q1 is 1); **and** the study participants are aware of the research question (Q2 is 1).

**E)  DATA COLLECTION METHODS**

**Strong:** The data collection tools have been shown to be valid (Q1 is 1); **and** the data collection tools have been shown to be reliable (Q2 is 1).

**Moderate:** The data collection tools have been shown to be valid (Q1 is 1); **and** the data collection tools have not been shown to be reliable (Q2 is 2) **or** reliability is not described (Q2 is 3).

**Weak:** The data collection tools have not been shown to be valid (Q1 is 2) **or** both reliability and validity are not described (Q1 is 3 and Q2 is 3).

**F) WITHDRAWALS AND DROP-OUTS - a rating of:**

**Strong:** will be assigned when the follow-up rate is 80% or greater (Q2 is 1).

**Moderate:** will be assigned when the follow-up rate is 60 – 79% (Q2 is 2) **OR** Q2 is 5 (N/A).

**Weak:** will be assigned when a follow-up rate is less than 60% (Q2 is 3) or if the withdrawals and drop-outs were not described (Q2 is 4).
